# Sustained and Microenvironment-Accelerated Release of Minocycline from Alginate Injectable Hydrogel for Bacteria-Infected Wound Healing

**DOI:** 10.3390/polym14091816

**Published:** 2022-04-29

**Authors:** Chengjia Xie, Qun Zhang, Zhao Li, Shaohua Ge, Baojin Ma

**Affiliations:** Department of Periodontology & Tissue Engineering and Regeneration, School and Hospital of Stomatology, Cheeloo College of Medicine, Shandong University & Shandong Key Laboratory of Oral Tissue Regeneration & Shandong Engineering Laboratory for Dental Materials and Oral Tissue Regeneration, Shandong University, Jinan 250012, China; xiecj_cherry@126.com (C.X.); zhangqunpku@126.com (Q.Z.); 15254276332@163.com (Z.L.)

**Keywords:** injectable hydrogel, alginate, microenvironment-accelerated release, antibacterial activity, wound healing

## Abstract

During wound healing, bacterial infection is one of the main limiting factors for the desired efficiency. Wound dressing-mediated antibiotics therapies could overcome this problem to a great extent due to sustained drug release and controllable dose. Here, we designed a kind of alginate injectable hydrogel loaded with minocycline (SA@MC) as a dressing for staphylococcus aureus-infected wound healing. SA@MC hydrogel possessed good injectability and can be injected by syringes. MC participated in the gel formation, causing the microstructure change based on the morphology characterization. The element mapping and FT-IR spectra further confirmed the successful loading of MC in SA hydrogel. Interestingly, MC was released more efficiently in a weakly alkaline condition (pH 7–8) than in a weakly acidic condition (pH 4–6) from SA@MC injectable hydrogel, which means that there is an accelerated release to respond to the weakly alkaline wound microenvironment. Meanwhile, SA@MC injectable hydrogel had high biocompatibility and excellent antibacterial activity due to the sustained release of MC. Further, in vivo experiment results demonstrated that SA@MC injectable hydrogel promoted staphylococcus aureus-infected wound healing efficiently. In summary, the injectable composite hydrogel can serve as an ideal dressing to prevent bacterial infection and promote wound healing.

## 1. Introduction

As the largest defensive barrier, the skin keeps the body from bacterial infection, water loss, and invasion of harmful substances [1,2]. Due to the direct contact with the surroundings, skin is easy to be injured and becomes one of the most vulnerable tissues [3]. The damaged skin will lose the ability to defend against bacterial invasion, leading to infection and inflammation, and impeding efficient wound repair by increasing cytokines and decreasing the growth factor [2,4]. Therefore, bacterial infection during wound repair attracts many public health concerns, and also brings an enormous medical and financial burden.

Until now, antibiotics, such as tetracycline, gentamicin, and minocycline, still are widely applied in clinics. Although antibiotic therapy is a milestone in infectious diseases treatment, the side effects, especially of systemic administration on skin allergies, digestive system, and genetic variability cannot be ignored [5,6]. Meanwhile, the overuse of antibiotics also causes the generation of drug resistance in bacteria, leading to the failure of antibiotic therapy [7,8]. To overcome the side effects and drug resistance generation during antibiotic therapy, strategies based on local and controllable administration have been developed, especially for bacteria-infected wound healing and periodontitis [9,10].

Varied wound dressings, such as foams, electrospun fibers, membranes, and hydrogels, have been widely used to protect skin from damage, promote wound healing, and reduce scar formation. Wound dressings possess two obvious intrinsic advantages for antibiotic therapy, localization of drug administration and controllable dose, which could efficiently eliminate the side effects and drug resistance generation. Compared with other kinds of wound dressings, hydrogels dressings can keep a moist microenvironment in the local wound, absorb tissue exudates, allow gas exchange, and relieve pain, which are beneficial to healing [11]. Among these, injectable hydrogels possess more advantages than non-injectable hydrogels, such as good filling capability, high loading capability, and efficient release during bacteria-infected wound healing, therefore attracting more and more attention [12].

Alginate is a naturally occurring anionic and hydrophilic polysaccharide that contains blocks of (1–4)-linked β-D-mannuronic acid (M) and α-L-guluronic acid (G) monomer. The conformations of the guluronate blocks provide strong coordination of the divalent ions. The G blocks of one polymer chain can bind to adjacent blocks using divalent cations, resulting in the formation of a gel structure. Sodium alginate (SA) is an anionic polymer as the sodium salt of alginate, which can form ionic bonds with cations. For example, it can form an ionic crosslinked gel with Ca^2+^, Ba^2+^, Sr^2+^, and Fe^2+^. Ca^2+^ is safe and inexpensive. Therefore, CaCl_2_ is the most commonly used ionic crosslinking agent. Due to the high biocompatibility, good biodegradation, and efficient bioactivity, SA hydrogel is considered to be an ideal candidate for wound dressing. Johnson et al. designed a kind of ibuprofen/SA hydrogel for accelerated burn wound healing by pressurized gas expanded liquid (PGX) technology [13]. Chen et al. constructed a kind of covalently antibacterial SA/chitosan/tetracycline hydrogel for efficient wound healing [14]. However, the phenomenon of burst release is the barrier to long-term antibacterial activity of hydrogel-mediated antibiotic therapy, which brings a risk of reinfection. For example, ~50% amoxicillin was released from N-carboxyethyl chitosan/oxidized hyaluronic acid-graft-aniline tetramer hydrogel within 24 h [15], ~50% doxycycline was released from hyaluronic acid-graft-dopamine/reduced graphene oxide hydrogel within 24 h [16], and 70% ibuprofen was released from PGX-alginate hydrogel within 10 h [13]. Similarly, the current SA-based hydrogel drug delivery systems usually demonstrated the burst release and failed to release drugs continuously. The burst release not only shortens the antibacterial period but also damages the normal cells and tissues due to the locally high concentration of antibiotics. Therefore, designing a kind of injectable and antibiotic-loaded SA hydrogel without a burst release is crucial for efficient bacteria-infected wound healing. Further, healthy skin tissue generally has a weakly acidic microenvironment (pH 4–6). However, after suffering damage or infection, the microenvironment in wound skin tissue significantly increases up to 7–8 [17]. How to maintain the efficient release of drugs in a weakly alkaline microenvironment is important to increase antibacterial efficiency.

Here, we constructed an injectable SA hydrogel containing minocycline (MC) (SA@MC) with good antibacterial activity, sustained release ability, and high cytocompatibility. MC plays roles both on Gram-positive and Gram-negative bacteria and exhibits broad-spectrum antibacterial properties. Importantly, MC can prevent cell death, which is helpful to promote wound healing [18]. The injectable ability, microstructure, porosity, and release profiles of SA@MC injectable hydrogels were characterized. The cytocompatibility and antibacterial ability were assessed in vitro, and the wound healing ability was confirmed by a bacteria-infected wound model in vivo.

## 2. Materials and Methods

### 2.1. Materials and Preparation of SA@MC Injectable Hydrogels

A 4 mL sodium alginate (molecular weight: 20–50 k; viscosity: 15–60 mpa·s; M:G = 1:1; Macklin, Shanghai, China) aqueous solution with a concentration of 5 wt.% was prepared, and then 20 mg MC (Minocycline hydrochloride, Macklin, Shanghai, China) was added. Then, 1 mL calcium chloride dihydrate (Macklin, Shanghai, China) aqueous solution with a concentration of 1 wt.% was added slowly for 10 min into the sodium alginate aqueous solution to form SA@MC4 injectable hydrogel (MC 4 mg/mL). SA, SA@MC1 (MC 1 mg/mL), and SA@MC2 (MC 2 mg/mL) injectable hydrogels were prepared based on the same procedures.

### 2.2. Characterization of SA@MC Injectable Hydrogels

The microstructures of SA@MC injectable hydrogels with different MC concentrations were observed by the scanning electron microscope (SEM, Pro G5, Phenom, Phenom world, Eindhoven, The Netherlands). Elements’ mapping images were recorded by Energy Dispersive Spectrometer equipped in the mentioned SEM. FT-IR spectra were measured by Bruker ALPHA II. Release profiles were recorded by microplate reader (CMax Plus, Molecular Devices, Shanghai, China) based on the absorption at 405 nm. The porosity was determined by (M1-M2) /M1, where M1 is the mass of a certain volume of hydrogel after swelling for 24 h and M2 is the mass after freeze-drying. The pore sizes of different SA hydrogels with different concentrations of MC were measured by SEM images (*n* = 6 per group). The unconfined compressive tests were conducted using an Electronic Strength Tester (AGS-X, SHIMADZU, Kyoto, Japan). Rheological measurement was performed by a rheometer (MCR 302, Anton Paar, Senso Tech, Graz, Austria).

### 2.3. Drug Release Kinetics

The release amount of MC was detected by using PBS solutions with different pH values. In brief, 1 mL of injectable hydrogel samples was immersed in 10 mL of release medias in a shaking water bath at 100 rpm and with a temperature of 37 °C, and at a predetermined time interval, 1 mL of release medium was collected and replaced with 1 mL of fresh PBS solution. The amount of drug release was then analyzed using a microplate reader at 405 nm for MC based on a calculated standard curve. The cumulative MC release percentages were determined by Mt/M0 × 100, where Mt is the total amount of MC released at time t and M0 is the initial amount of MC in the hydrogel.

### 2.4. Cytocompatibility Evaluation of SA@MC Injectable Hydrogels

The 1 mL of different concentrations of SA@MC were added in to a 15 mL sterile centrifuge tubes with another 10 mL MEM-α medium (VivaCell, Shanghai, China). Then, the centrifuge tubes were placed in a 37 °C constant temperature at a shaking speed of 100 rpm for 24 h. The obtained extracts were used to culture cells. Human dermal fibroblasts (HDFs) were obtained from Wu’s laboratory. HDFs were cultured in MEM-α medium supplemented with 10% fetal bovine serum (BioInd, Kibbutz Beit Haemek, Israel) and antibiotics (100 U/mL penicillin G, 100 mg/mL streptomycin, Sigma-Aldrich, St. Louis, MO, USA). HDFs were seeded in a 96-well plate at the density of 3000 cells/well. After being cocultured with the extracts from the SA@MC injectable hydrogels, the cell viability and state were evaluated by CCK-8 Assay Kit (Solarbio, Beijing, China) and LIVE/DEAD Viability Kit (BestBio, Shanghai, China), respectively. For the CCK-8 assay, after being incubated for 1, 2, and 3 days, 100 μL MEM-α containing a 10% CCK-8 reagent was added into each well. After incubation at 37 °C for 2 h, optical density (OD) was recorded by a microplate reader at 450 nm. For the live/dead assay, cells were cocultured with the extracts from SA@MC injectable hydrogels for 1 day, then 200 μL MEM-α containing the live/dead staining reagent was added into each well for 20 min, then images were taken by fluorescence microscope (IX53, Olympus, Tokyo, Japan). For cytoskeleton staining, cells were fixed with 4% paraformaldehyde for 10 min, and rinsed with PBS for 3 times (5 min each time). A 0.5% Triton X-100 was added to each well for 10 min to permeate the cell membrane. After being rinsed by PBS, 1% BSA was added to seal at room temperature for 1 h. FITC-labeled Phalloidine (dilution ratio: 1:200, Sigma-Aldrich) was added and incubated overnight at 4 °C, avoiding light. The next day, the dyed solution was sucked out, and DAPI work solution (Solarbio) was added to each well for 5 min before rinsing. An inverted fluorescence microscope (IX53, Olympus) was employed to observe the cell viability and cytoskeleton.

### 2.5. Antibacterial Activity Evaluation

For the inhibiting ring test, 100 μL SA@MC and SA injectable hydrogels were injected into a tubular container, which was 4 mm in both height and diameter, respectively. A single colony of Staphylococcus aureus with an area of 2 cm^2^ were dipped with a sterile cotton swab and put into 10 mL sterile physiological saline. After being mixed evenly, the concentration of the bacterial solution was adjusted with physiological saline until OD = 0.257 at 600 nm (~10^8^ CFU/mL), and then was diluted into 10^4^ CFU/mL. The 200 μL 10^4^ CFU/mL bacterial solution was coated on the nutrient agar plate. After the bacteria solution was dried, SA@MC hydrogel was placed on the plate, and incubated at 37 °C. The inhibiting ring was analyzed after 10 h. Polyethyleneimine (PEI) was used as a positive control.

The 4 mL of SA or SA@MC injectable hydrogels were centrifugated at 6000 rpm for 2 min to place the gel at the bottom of the tubes. The 15 mL of 10^6^ CFU/mL bacterial solution was added and coculture for 5 h before the bacteria solution was collected. For the colony counting test, 200 μL 10^4^ CFU/mL of the above bacterial solution were coated on the nutrient agar plates with a coating stick and incubated for 10 h at 37 °C. For the live/dead assay, resuspending the bacteria solution with a 200 μL live/dead Bacterial Staining (BestBio) work solution was prepared, and an inverted fluorescence microscope (IX53, Olympus) was employed to observe the state of the bacteria. A part of the above bacterial solution was resuspended with Glutaraldehyde 4% (EM Grade, Aladdin, Shanghai, China) for 12 h at 4 °C. Then, the gradient elution was performed for the bacteria with 35%, 50%, 65%, 80%, 95%, and 100% ethanol for 15 min each time. Then, bacteria were collected by centrifugation at 8000 rpm for 8 min, and were resuspend with absolute ethyl alcohol for SEM characterization.

### 2.6. In Vivo Wound Healing in a Full-Thickness Skin Defect Model

Animal studies were conducted in accordance with the guidelines of the Care and Use of Laboratory Animals of the Chinese Science and Technology Ministry and this study was approved by the Medical Ethics Committee of the School of Stomatology, Shandong University, Jinan, China (Protocol Number: NO.20220206). All surgical procedures were performed under pentobarbital sodium anesthesia and all efforts were made to minimize the suffering the animals might experience.

The in vivo wound healing was carried out by a full-thickness skin defect model. Male Wistar rat (250–300 g, 6–8 weeks age) were employed in this study. All rats were divided into 3 groups randomly, including the control group, SA hydrogel group, and SA@MC hydrogel group. The rats were anesthetized by intraperitoneal injection of chloral hydrate (0.3 mg/kg), then all rats were shaved in the dorsal region between tail and neck. Full thickness skin round wounds with 6 mm diameter were created by needle biopsy, and 20 μL bacterial solution (10^8^ CFU/mL) was added in the wound. For the control group, the wounds were added with 20 μL of PBS. For the hydrogel groups, 20 μL of SA hydrogel and SA@MC4 hydrogel was applied on the wounds respectively. Pictures of the rat wounds were taken at 1 d, 3 d, 6 d, and 12 d. For evaluation of inflammation in the wound area, samples were collected on the 6th day. After collection, samples were fixed with 4% paraformaldehyde for 24 h, then embedded in paraffin and cross sectioned to 4 μm thickness slices. All slices were stained with Haematoxylin-Eosin (Solarbio) and photo-captured by microscope (IX53, Olympus).

### 2.7. Statistical Analyses

All quantitative results are reported as mean ± standard deviation (SD) obtained from at least three independent studies. The statistical differences were calculated using a two-tailed Student’s *t*-test or analysis of variance (ANOVA), as appropriate. Statistical differences were defined as * *p* < 0.05, ** *p* < 0.01, and *** *p* < 0.001.

## 3. Results and Discussion

### 3.1. Injectable Property of SA@MC Injectable Hydrogels

**Figure 1:**
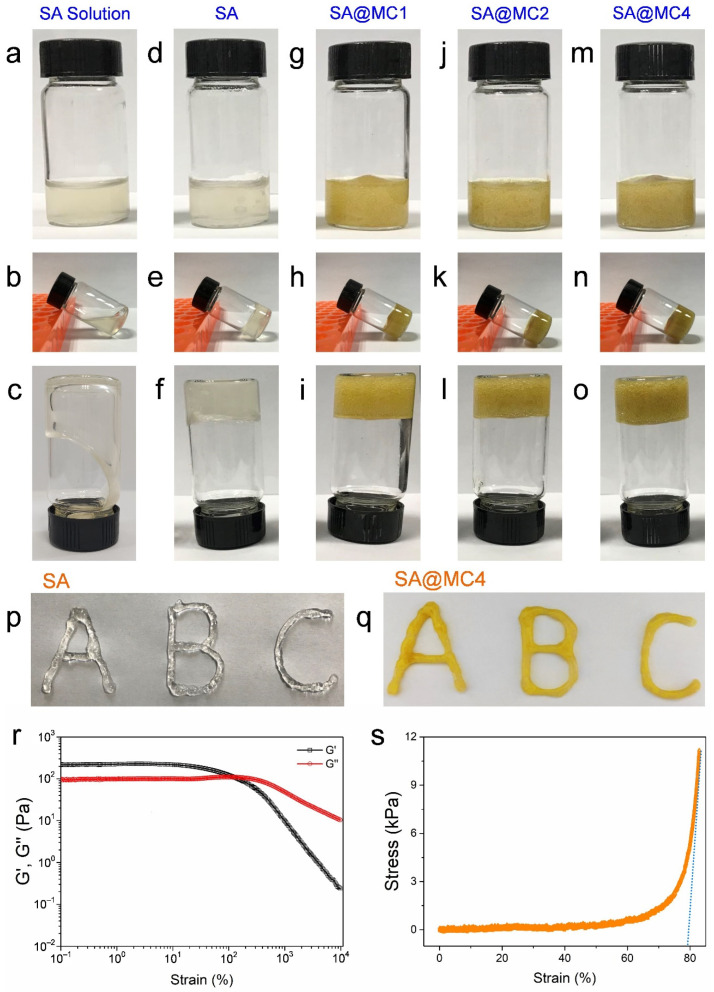
Injectable property of SA@MC hydrogel. (**a**–**c**) SA solution before Ca^2+^ crosslinking; (**d**–**f**) SA hydrogel; (**g**–**i**) SA@MC1 hydrogel; (**j**–**l**) SA@MC2 hydrogel; (**m**–**o**) SA@MC4 hydrogel; (**p**,**q**) the injectable property of SA and SA@MC4 hydrogels; (**r**) the G′ and G″ of the SA@MC4 on the strain amplitude sweep (c = 0.1–10,000%) at a fixed frequency (f = 1 Hz); (**s**) the tensile stress–strain curve of SA@MC4 injectable hydrogel.

Firstly, the injectable property of the SA@MC hydrogel was assessed. Before Ca^2+^ crosslinking, the SA solution was fluid and failed to gel (Figure 1a–c). After Ca^2+^ crosslinking, the SA gelled and lost its fluid ability (Figure 1d–f). The loading of MC did not change the hydrogel formation, and the yellow color became darker with the increased concentration (Figure 1g–o). Therefore, the SA@MC hydrogel was prepared successfully. To confirm the injectable property, we used syringes loaded with SA and SA@MC4 hydrogel, respectively, to write the letters of ABC (Figure 1p,q). Further, the storage modulus (G′) and loss modulus (G″) changes were recorded (Figure 1r). Both G′ and G″ remained unchanged on small strain (0.1–10%), but decreased when the strain was larger than a critical value (10% for G′ and 200% for G″) due to the rupture of the hydrogel network. At the strain of 127%, the G′ curve intersected with G″ curve, indicating that the hydrogel transformed from a solid state to fluid state. Meanwhile, the unconfined compressive test was conducted using an Electronic Strength Tester (Figure 1s). The hydrogel samples were cut into cylindrical shapes and compressed to 83% in order to measure the compressive modulus. It is found that the compression modulus of the SA@MC4 injectable hydrogel is not a constant value, which increases with compression strain. When the strain is 83%, E is ~0.29 MPa, tending to be constant thereafter. The above results demonstrated that SA@MC4 hydrogel both possessed a good injectable ability, which is helpful in acting as an efficient wound dressing.

### 3.2. Characterization of SA@MC Injectable Hydrogels

**Figure 2:**
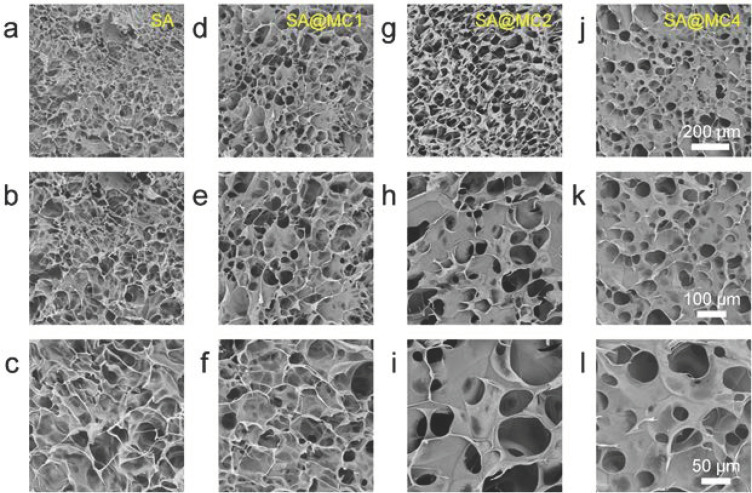
Microstructure of SA@MC hydrogels. (**a**–**c**) SA hydrogel; (**d**–**f**) SA@MC1 hydrogel; (**g**–i) SA@MC2 hydrogel; (**j**–**l**) SA@MC4 hydrogel.

To observe the microstructure, SA injectable hydrogels with different MC concentrations were observed by SEM after freeze-drying. The SA injectable hydrogel demonstrated a typical morphology and porous structure, similar to the dopamine-modified gelatin injectable hydrogel and chitosan/pectin injectable hydrogel (Figure 2a–c) [19,20]. The addition of MC can obviously change the morphology and structure. SA injectable hydrogel had small-size pores and flakes. Interestingly, SA@MC injectable hydrogels possessed big-size pores and flakes (Figure 2d–l). The pore sizes of different SA@MC hydrogels were measured based on SEM images (*n* = 6 per group) and were 26.4 ± 8.2 μm (SA), 43.9 ± 8.6 μm (SA@MC1), 56.8 ± 3.9 μm (SA@MC2), and 49.6 ± 8.1 μm (SA@MC4), respectively. The results demonstrated that MC participated in the gel formation, which should attribute to the special chemical structure of MC. MC has one negative charge from the deprotonation of the hydroxyl group at C3 and one positive charge from the protonated dimethylamine group at C4 [21,22]. The hydroxyl group at C3 with one negative charge can interact with metal ions and the protonated dimethylamine group at C4 with one positive charge can interact with SA molecules by electrostatic attraction. The interaction between MC and SA injectable hydrogel is helpful to increase the loading capacity and inhibit the burst release.

**Figure 3:**
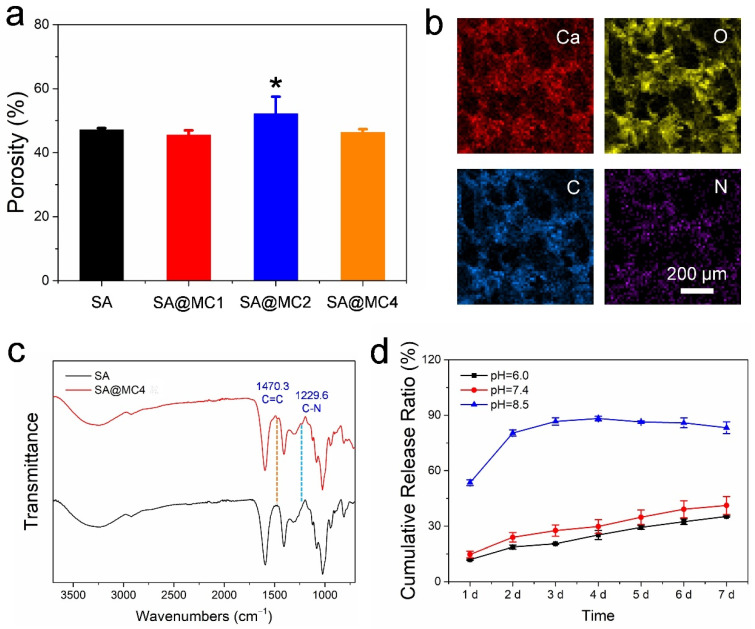
(**a**) The porosity of SA@MC injectable hydrogels at different concentrations of MC; (**b**) element mapping of SA@MC4 injectable hydrogel; (**c**) FT-IR spectra of SA and SA@MC4 injectable hydrogels; (**d**) Release profiles of SA@MC4 injectable hydrogel at different pH values. * *p* < 0.05 vs. the control group.

The porosity plays an obvious effect on the drug release from the hydrogel, and the low porosity can limit drug release and also easily cause the burst release phenomenon [23]. The porosities of SA@MC injectable hydrogels were measured. Based on the results (Figure 3a), the loading of MC almost did not change the porosity and even increased the porosity slightly when the concentration was 2 mg/mL. The high porosity can improve the antibacterial efficiency by regulating the efficient release of MC from SA@MC injectable hydrogels. To confirm the distribution of MC, elements’ mapping images were obtained. Because the N element only exists in MC, its distribution can represent the MC. The distributions of Ca, O, C, and N were consistent in the SA@MC4 injectable hydrogel (Figure 3b), which means that MC was distributed evenly in SA@MC injectable hydrogels. Further, the FT-IR spectra of SA and SA@MC4 injectable hydrogel were obtained (Figure 3c). The SA injectable hydrogel demonstrated typical peaks (such as the deprotonated carboxyl group at ~1407 and ~1593 cm^−1^) [24,25,26]. The peaks of C=C in benzene ring at ~1470 cm^−1^ and C–N at 1230 cm^−1^ appeared in the SA@MC4 injectable hydrogel spectrum, meaning the successful loading of MC, which is consistent with the result of element mapping.

The MC is easily soluble in 5 wt.% sodium alginate at 4 mg/mL without any precipitation. Therefore, in our study, the encapsulation efficiency (EE%) is 100% and the loading degree (LD%) is 0.38%. Due to the weakly alkaline microenvironment of skin tissue after suffering damage or infection, the release profiles were assessed in PBS at pH = 7.4 and pH = 6.0 (simulating the weakly acid microenvironment of healthy skin tissue) (Figure 3d). Interestingly, MC demonstrated more efficient release in the weakly alkaline microenvironment than in the weakly acid microenvironment. The increased release of MC in the weakly alkaline microenvironment can bring higher antibacterial efficiency. The relatively strong alkaline microenvironment (pH = 8.5) was used as a positive control. It is clear that there was a burst release (release ratio ~53.5% within 24 h). It is well known that SA hydrogels are stable in the acid environment and easy to be degraded in the alkaline environment [27]. Therefore, the SA@MC4 injectable hydrogel demonstrated an accelerated release of MC in response to the microenvironment in the bacteria-infected wound.

### 3.3. Biocompatibility of SA@MC Injectable Hydrogels

**Figure 4:**
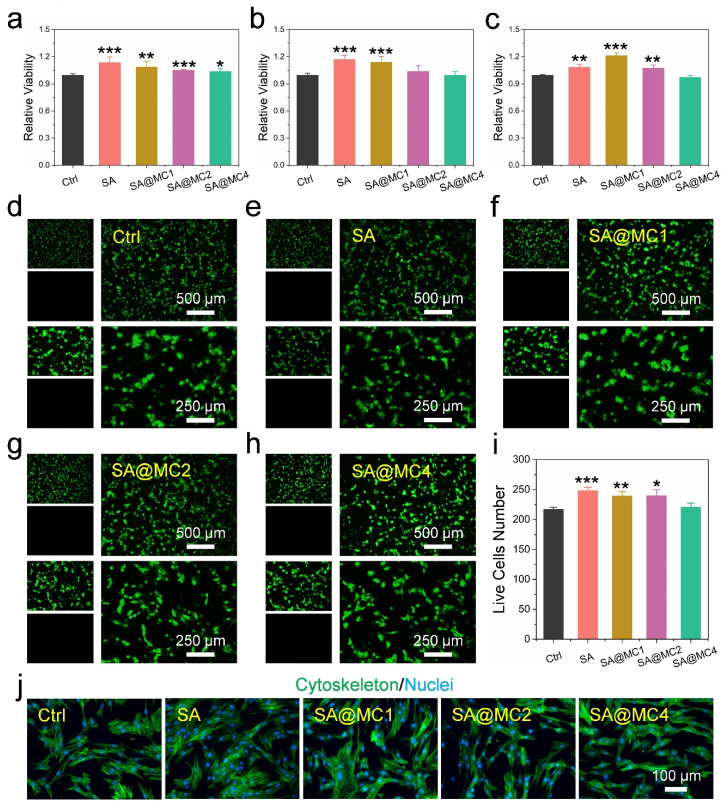
Biocompatibility assessment of SA@MC injectable hydrogels. (**a**–**c**) Cell viability of HDFs cocultured with the extracts from SA@MC injectable hydrogels at different concentrations of MC after different times; (**d**–**h**) live/dead staining of HDFs cocultured with the extracts from SA@MC injectable hydrogels at different concentrations of MC after 24 h; (**i**) live cell number statistics; (**j**) cytoskeleton/nuclei staining of HDFs cocultured with the extracts from SA@MC injectable hydrogels at different concentrations of MC after 24 h. * *p* < 0.05, ** *p* < 0.01, and *** *p* < 0.001 vs. the control group.

To assess the biocompatibility of SA@MC injectable hydrogels, a CCK-8 viability measurement and live/dead staining were performed. The extracts from SA@MC injectable hydrogels with different MC concentrations were cocultured with human dermal fibroblasts (HDFs) for 24 h, 48 h, and 72 h, respectively. After 24 h, the cells’ viabilities in all groups were slightly higher than in the control group (Figure 4a). After 48 h, cells in SA and SA@MC1 groups demonstrated higher viability than the control group, and there were no significant differences between SA and SA@MC2 groups, SA and SA@MC4 groups, respectively (Figure 4b). After 72 h, cells in SA, SA@MC1, SA@MC2 groups possess higher viability, and there was no significant difference between SA and SA@MC4 groups (Figure 4c). The live/dead staining results after being cocultured for 24 h also demonstrated similar results. There were almost no dead cells in all groups (Figure 4d–h). Meanwhile, SA, SA@MC1, and SA@MC2 injectable hydrogels promoted cell proliferation slightly based on live cell numbers’ statistics, and there was no significant difference between SA and SA@MC4 group (Figure 4i). Further, cytoskeleton staining was performed to observe cells’ morphology (Figure 4j). Cells in all groups exhibited the typical shape of HDFs, meaning that SA@MC injectable hydrogels have no effect on cell spreading. Therefore, based on the above results, SA@MC injectable hydrogels possessed high biocompatibility. To obtain a better antibacterial efficiency, SA@MC4 injectable hydrogel was used in the following antibacterial and in vivo experiments.

### 3.4. Antibacterial Activity of SA@MC Injectable Hydrogels

**Figure 5:**
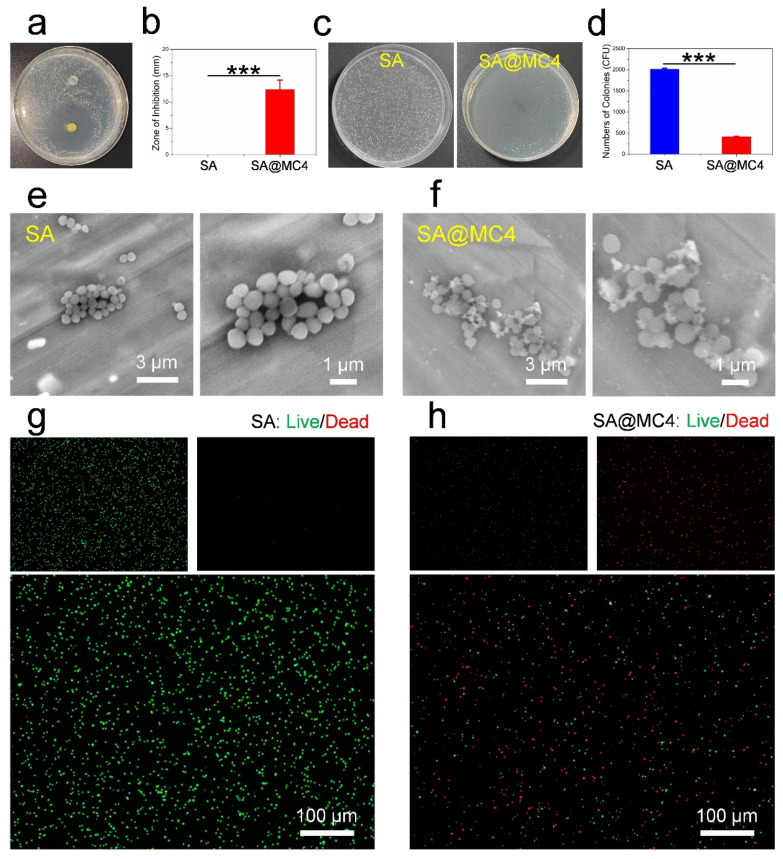
The antibacterial activity assessment of SA@MC injectable hydrogels. (**a**,**b**) Inhibition ring; (**c**,**d**) colony counting; (**e**,**f**) SEM images and (**g**,**h**) live/dead staining of bacteria after being cocultured with SA and SA@MC4 injectable hydrogels. *** *p* < 0.001 vs. the control group.

The antibacterial efficiency of SA@MC4 injectable hydrogel was assessed by the inhibition ring and colony counting. There was no inhibition ring around the SA injectable hydrogel, and an obvious inhibition ring with a diameter of ~12 mm appeared around the SA@MC4 injectable hydrogel (Figure 5a,b), similar with the results (~9 mm, 10 μL; ~14 mm, 20 μL) of PEI as a positive control. After being cocultured with SA injectable hydrogel, the bacteria formed many (~2011) big colonies. However, only a small number (~412) of small colonies formed after the bacteria cocultured with SA@MC4 injectable hydrogel. Further, after being cocultured with SA injectable hydrogel, the bacteria kept integral morphology, and a lot of fragments were observed after being cocultured with SA@MC4 injectable hydrogel, meaning that many bacteria died. To further confirm the state of the bacteria, live/dead staining was performed. SA injectable hydrogel did not cause bacteria death. However, many dead bacteria appeared after being cocultured with SA@MC4 injectable hydrogel. Therefore, SA@MC4 injectable hydrogel possessed good antibacterial activity.

### 3.5. Wound Healing Ability In Vivo of SA@MC Injectable Hydrogels

**Figure 6:**
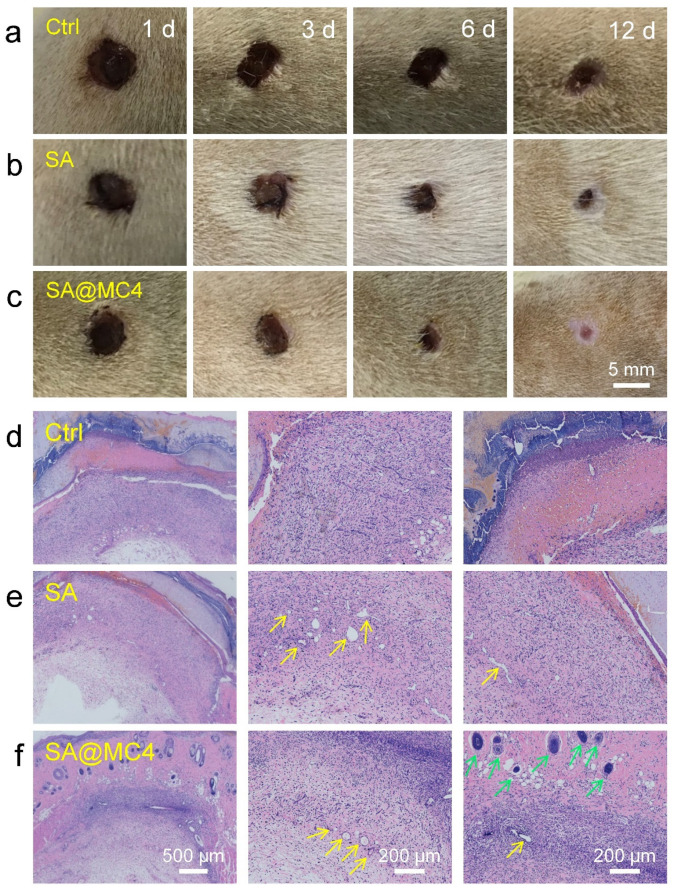
Wound healing ability assessment. (**a**–**c**) Photographs of wounds on the 1st, 3rd, 6th, and 12th day after treatment with PBS, SA hydrogel, and SA@MC4 hydrogels; (**d**–**f**) histomorphological evaluation of wound regeneration after treatment with PBS, SA hydrogel and SA@MC4 hydrogels on the 6th day (blood vessels: yellow arrows; hair follicles: green arrows).

The wound healing performance of the hydrogels was evaluated in a bacteria-infected full-thickness skin defect model. The wound contraction areas changes were displayed after being treated with PBS, SA, and SA@MC4 injectable hydrogels on the 1st, 3rd, 6th, and 12th day (Figure 6a–c). The SA@MC4 hydrogel group demonstrated fast wound contraction speed during the whole treatment period. On the 6th day, all groups demonstrated wound area contraction to some extent, and the SA@MC4 hydrogel groups demonstrated a smaller wound area than PBS and SA hydrogel groups. Based on H&E staining results (Figure 6d–f), fibroblasts migrated to the wound site in all groups and SA hydrogel and SA@MC4 hydrogel groups formed a layer of complete epithelium. The SA@MC4 hydrogel group exhibited more regular connective tissue. In addition, compared with other groups, more new blood vessels and hair follicles formed in the SA@MC4 hydrogel group. Therefore, the maximum amount of mature hair follicles and blood vessels, well-proliferated fibroblast, and thickened epidermis demonstrated that SA@MC4 hydrogel had the best wound healing effect among the all groups.

## 4. Conclusions

In summary, we designed a kind of alginate injectable hydrogel loaded with minocycline (SA@MC) as a dressing for bacteria-infected wound healing. Microstructure change demonstrated that MC participated in gel formation, bringing high loading capacity and sustained release ability. The element mapping and FT-IR spectra further confirmed the successful loading of MC in the SA hydrogel. Interestingly, MC was released more efficiently in a weakly alkaline condition than in a weakly acidic condition from SA@MC injectable hydrogel. Therefore, a microenvironment-response accelerated release of MC was achieved by responding to the weakly alkaline wound microenvironment, bringing an efficient antibacterial effect. Meanwhile, the SA@MC injectable hydrogel had high biocompatibility to confirm the biosecurity during applications. Further, the in vivo experiment results demonstrated that SA@MC injectable hydrogel efficiently promoted bacteria-infected wound healing. Based on the good antibacterial activity and high biocompatibility, the injectable composite hydrogel can serve as an ideal dressing to prevent bacterial infection and promote wound healing.

## Data Availability

Not applicable.
